# Advancements in osteosarcoma management: integrating immune microenvironment insights with immunotherapeutic strategies

**DOI:** 10.3389/fcell.2024.1394339

**Published:** 2024-06-10

**Authors:** Hang Liang, Min Cui, Jingyao Tu, Xinyi Chen

**Affiliations:** ^1^ Department of Orthopedics, Union Hospital, Tongji Medical College, Huazhong University of Science and Technology, Wuhan, China; ^2^ Department of Oncology, Tongji Hospital, Tongji Medical College, Huazhong University of Science and Technology, Wuhan, China

**Keywords:** osteosarcoma, tumor immune microenvironment, immunotherapy, tumor microenvironment modulators, cell-based therapies, immune checkpoint inhibitors

## Abstract

Osteosarcoma, a malignant bone tumor predominantly affecting children and adolescents, presents significant therapeutic challenges, particularly in metastatic or recurrent cases. Conventional surgical and chemotherapeutic approaches have achieved partial therapeutic efficacy; however, the prognosis for long-term survival remains bleak. Recent studies have highlighted the imperative for a comprehensive exploration of the osteosarcoma immune microenvironment, focusing on the integration of diverse immunotherapeutic strategies—including immune checkpoint inhibitors, tumor microenvironment modulators, cytokine therapies, tumor antigen-specific interventions, cancer vaccines, cellular therapies, and antibody-based treatments—that are directly pertinent to modulating this intricate microenvironment. By targeting tumor cells, modulating the tumor microenvironment, and activating host immune responses, these innovative approaches have demonstrated substantial potential in enhancing the effectiveness of osteosarcoma treatments. Although most of these novel strategies are still in research or clinical trial phases, they have already demonstrated significant potential for individuals with osteosarcoma, suggesting the possibility of developing new, more personalized and effective treatment options. This review aims to provide a comprehensive overview of the current advancements in osteosarcoma immunotherapy, emphasizing the significance of integrating various immunotherapeutic methods to optimize therapeutic outcomes. Additionally, it underscores the imperative for subsequent research to further investigate the intricate interactions between the tumor microenvironment and the immune system, aiming to devise more effective treatment strategies. The present review comprehensively addresses the landscape of osteosarcoma immunotherapy, delineating crucial scientific concerns and clinical challenges, thereby outlining potential research directions.

## 1 Introduction

Osteosarcoma, the most prevalent bone tumor among children and adolescents, is characterized by the formation of osteoid and immature bone at the metaphysis and diaphysis of long bones (Mirabello et al., 2009). Accounting for approximately 20% of all bone malignancies, this highly aggressive tumor is renowned for its aggressive proliferation and infiltration capabilities, frequently leading to rapid invasion of adjacent bone tissue and structures, subsequently resulting in significant bone destruction and functional impairment. Given its propensity for early pulmonary metastasis and aggressive characteristics, the treatment of osteosarcoma presents considerable challenges (Durfee et al., 2016; Harrison et al., 2018). Although surgical resection combined with neoadjuvant chemotherapy, involving agents such as doxorubicin, cisplatin, methotrexate, and ifosfamide, is proven to be effective in managing primary lesions, there is a notable absence of efficacious treatments for metastatic osteosarcoma (Ta et al., 2009). Remarkably, while the five-year survival rate for patients with localized osteosarcoma exceeds 78%, this rate falls to 25% for metastatic or recurrent osteosarcoma (Gaspar et al., 2018), highlighting the urgent necessity for innovative therapeutic approaches for osteosarcoma.

Recent research has increasingly focused on the tumor microenvironment (TME) of osteosarcoma, aiming to elucidate the tumor’s heterogeneity, progression, and metastasis, thereby contributing insights into this highly aggressive tumor. Osteosarcoma proliferates within a complex, dynamic bone microenvironment comprising osteoblasts, stromal cells, vascular cells, immune cells, and mineralized extracellular matrix (ECM) (Alfranca et al., 2015). Crucial to tumor progression are the intricate interactions among osteosarcoma cells and the adjacent microenvironment, influencing tumor progression, apoptosis, invasion, metastasis, angiogenesis, pre-metastatic niche formation, and therapeutic response (Tsagozis et al., 2020; Zhu et al., 2022). Characterized by considerable immune cell infiltration, the osteosarcoma-adjacent tissue manifests an intricate immune microenvironment, facilitating the intraosseous proliferation and persistence of osteosarcoma cells in an immunosuppressive background (Pierrevelcin et al., 2022; Somaiah et al., 2022). The components of this microenvironment are predominantly categorized into cellular elements, including tumor-associated macrophages (TAMs), tumor-associated neutrophils (TANs), myeloid-derived suppressor cells (MDSCs), mast cells (MCs), T cells, B cells, natural killer (NK) cells, dendritic cells (DCs), and non-cellular components such as mesenchymal stem cells (MSCs) and circulating tumor cells (CTCs), all of which contribute to interactions with the immune system, thereby promoting suppressive networks (Zheng Y. et al., 2018). Additionally, research involves the complement system and exosomes, recognized for their specific immune responses (Mathieu et al., 2019). The immunosuppressive microenvironment’s intensity correlates with increased activity of molecules including indoleamine 2,3-dioxygenase (IDO), programmed cell death protein 1 (PD-1), interleukin-10 (IL-10), transforming growth factor-beta (TGF-β), vascular endothelial growth factor (VEGF), and signal transducer and activator of transcription 3 (STAT3), primarily mediated by MDSCs, TAMs, and regulatory T lymphocytes (Tregs) with immunosuppressive characteristics (Xie et al., 2022; Kuo et al., 2023). Therefore, it is essential to elucidate the interactions between osteosarcoma cells and immune cells to develop the effectiveness of immunotherapies.

Recent trials have explored the efficacy of PD-1 and CTLA-4 targeted immunotherapies in osteosarcoma (Wang et al., 2016; Ratti et al., 2017; Hennessy et al., 2021). Subsequent clinical studies, however, have indicated a restrained response to anti-PD-1 immunotherapy among a limited patient cohort. Furthermore, the efficacy of anti-CTLA-4 immunotherapy in osteosarcoma clinical treatment remains ambiguous (Thanindratarn et al., 2019). The interactions among cancer, immune, and stromal cells in osteosarcoma lead to the development of an immunosuppressive TME, thereby facilitating cancer cells’ evasion of immune responses and contributing to the heterogeneity of the tumor, which is associated with treatment resistance and differences in patient responses (Suehara et al., 2019). Comprehending the heterogeneity of cancer cells and the dynamic tumor immune microenvironment might identify novel therapeutic targets for the treatment of osteosarcoma.

In conclusion, a profound exploration of the tumor’s biological characteristics and cellular interactions within its microenvironment is essential for the development of therapeutic strategies for osteosarcoma, particularly crucial for the management of metastatic osteosarcoma. This review comprehensively explores osteosarcoma from aspects including its pathophysiological characteristics, mechanisms of development, along with the contribution of immune cells in the tumor’s microenvironment. A novel approach is proposed to improve treatment efficacy by modulating the immune cells within the TME. The current review is innovative in its comprehensive investigation of the osteosarcoma immune microenvironment and its role in oncological progression, suggesting therapeutic strategies incorporating immune checkpoint inhibitors (ICIs), TME modulators, and cytokine therapy. These approaches focus on the intricate immune microenvironment of osteosarcoma, providing innovative treatment perspectives and methods, particularly for complicated cases of metastasis.

## 2 Immune cells

### 2.1 Macrophages

In osteosarcoma, TAMs are identified as predominant components, comprising approximately half of the tumor mass, an observation that highlights their substantial role within the tumor’s immune microenvironment (Huang et al., 2021). Demonstrating considerable flexibility and heterogeneity, these cells impact tumor characteristics by either promoting or suppressing tumor functions, dependent on tumor type and specific TME interactions (Mantovani et al., 2017; Kielbassa et al., 2019). TAMs manifesting M1-like features are generally associated with anti-tumorigenic effects, capable of inducing tumor cell apoptosis and enhancing immune responses, whereas most, exhibiting an M2-like phenotype with immunosuppressive properties, predominantly facilitate angiogenesis, promote extracellular matrix invasion, and enable immune evasion, thereby contributing to tumor advancement and metastasis (Noy and Pollard, 2014; Franklin and Li, 2016). Such dichotomy highlights the imperative to comprehend the regulatory mechanisms controlling macrophage polarization and the subsequent roles in osteosarcoma.

Within osteosarcoma, the role of TAMs is essential, notably in tumor progression and the advancement of metastasis. Han et al. indicated that in osteosarcoma, M2-type TAMs enhance the function of TIM-3(+)PD-1(+) T cells, thereby intensifying T cell immunosuppression within the TME (Han et al., 2016). Additionally, the eradication of these TAMs enhances T cell proliferation and pro-inflammatory cytokine secretion, according to findings from Uehara et al., metformin treatment stimulates a conversion in TAMs from M2 to M1 phenotype, as indicated by elevated IL-12 and TNF-α levels, MHC class II upregulation, and CD206 downregulation, indicating a potential transition of TAMs from tumor-enhancing to tumor-restraining roles following metformin administration (Uehara et al., 2019). Li et al. indicated that ZIM3 in osteosarcoma cells induces CCL25 expression, as a result, leading M2-type TAMs to pulmonary metastatic nodules, thus promoting metastatic proliferation and highlighting the adverse impact of TAM infiltration in osteosarcoma (Li et al., 2023). Lin et al. elucidated that in osteosarcoma, MerTK-mediated phagocytosis promotes macrophage M2 polarization and PD-L1 expression via the p38/STAT3 pathway, intensifying immune tolerance and tumor progression, emphasizing the adverse impact of M2 polarization in increasing tumor aggressiveness (Lin et al., 2022). Liu et al. indicated that miR-221-3p, derived from M2-type TAMs, enhanced osteosarcoma cell proliferation and invasion via the JAK2/STAT3 pathway, targeting SOCS3, thereby exacerbating tumor malignancy (Liu et al., 2021). Cheng et al. highlighted the impact of osteosarcoma-derived exosomes in macrophage M2-type polarization mediated by Tim-3, along with their release of cytokines, particularly IL-10, TGF-β, and VEGF, facilitating increased cell migration, invasion, EMT, and lung metastasis (Cheng et al., 2021). Collectively, TAMs’ functions and polarization in the osteosarcoma microenvironment critically influence tumor progression. Therapeutic approaches such as the utilization of the CSF1/CSF1R inhibitor Pexidartinib (PLX3397) have shown promise in counteracting M2-type TAM polarization, thereby mitigating its tumor-promoting effects and demonstrating the potential for tumor growth and metastasis suppression through TAM polarization modulation (Fujiwara et al., 2021). Ségaliny et al. demonstrated that IL-34, with its expression upregulated in osteosarcoma, markedly enhances angiogenesis and recruits M2-type macrophages, a circumstance associated with enhanced M2-TAM infiltration, thereby emphasizing the role of M2-TAMs in the advancement of osteosarcoma (Segaliny et al., 2015). This finding is consistent with Zhou et al.’s research, which employed single-cell RNA analysis across different phases of osteosarcoma progression, identifying critical gene expression patterns in tumor cells and the surrounding microenvironment, with particular emphasis on variations in immune cells, and detected substantial infiltration of pro-inflammatory FABP4+ macrophages in lung metastatic osteosarcoma (Zhou et al., 2020a). Additionally, Wolf-Dennen et al. revealed that exosomes released by metastatic osteosarcoma cells initiate a conversion in alveolar macrophages towards a tumor-promoting M2 phenotype by activating IL10, TGFB2, and CCL22 mRNA, a process correlated with diminished capability in eradicating apoptotic cells and tackling tumor cells, thereby contributing to increased immune suppression and tumor progression in osteosarcoma (Wolf-Dennen et al., 2020). Through scRNA-Seq analysis of osteosarcoma lung metastases, Zheng et al. established that in tumor metastasis, TAMs predominantly manifest an immunosuppressive M2 phenotype, with PD-1 expression impeding phagocytic function and advancing tumor metastasis and progression (Zheng C. et al., 2024). Chen et al. identified that TIPE1 mitigates tumor growth by reducing macrophage infiltration, suggesting a potential new target for osteosarcoma treatment (Chen et al., 2019). Han et al. observed that osteosarcoma-associated macrophages activate the COX-2/STAT3 axis to enhance tumor metastasis and invasion, with COX-2 inhibition reducing metastasis (Han et al., 2019). Studies also demonstrated that IL13 and IL4 induce M2-phenotype macrophage polarization in the osteosarcoma microenvironment, impacting tumor cell invasiveness and metastatic capability, with MMP12 and other factors secreted by M2-phenotype macrophages playing a crucial role in osteosarcoma dissemination (All-Trans Retinoic Acid Prevents, 2020). Similarly, osteosarcoma macrophages were observed to secrete exosomes with lncRNA LIFR-AS1, considerably enhancing tumor cell growth and invasiveness via the miR-29a/NFIA pathway, and simultaneously reducing the frequency of apoptosis (Zhang H. et al., 2021). Furthermore, the application of TLR9 agonists in osteosarcoma treatments has been effective in constraining tumor growth by reducing infiltration of M2-type macrophages (Cascini et al., 2023). In Ewing’s sarcoma treatment, liposomal chloroquine and trabectedin effectively reduced M2-phenotype macrophages, thereby enhancing the effectiveness of oncolytic virotherapy and further confirming the crucial role of M2-phenotype macrophages in tumor progression (Denton et al., 2018). Nirala et al. elucidated that upregulation of microRNA 17/20a by the MYC gene leads to decreased levels of macrophage colony-stimulating factor 1 (CSF1), culminating in a reduced macrophage population within the TME, potentially influencing osteosarcoma progression and metastasis (Nirala et al., 2023). Similarly, the study by Zhang et al. on microRNA let-7a demonstrated its suppressive effect on Ewing’s sarcoma’s malignant characteristics through the inhibition of macrophage infiltration, emphasizing the adverse influence of TAMs in tumor advancement (Zhang et al., 2016). Additionally, Chim et al.'s research in the osteosarcoma microenvironment determined that TAMs exacerbate inflammation and enhance resistance to chemotherapeutic drugs, indicating an adverse effect of these macrophages on tumor progression (Chim et al., 2023). Yan et al. observed that exosomes from TAMs, mediated through exosome-let-7a, advance osteosarcoma metastasis, emphasizing TAMs’ significant role in advancing osteosarcoma (Yan et al., 2023). In addition, studies indicated that curcumin enhanced cisplatin’s (CDDP) anti-tumor effect by inhibiting M2-type macrophage polarization, suggesting a correlation between M2-type macrophage infiltration and tumor chemotherapy resistance (Wang J. et al., 2022). Li et al.’s study on osteosarcoma-derived microparticles (T-MPs) indicated initiation of macrophage polarization towards the M2 phenotype through the TBK1-STAT6 pathway, and manipulation of osteosarcoma cell conduct via the CCL18/STAT3 signaling pathway, thereby enhancing migration ability and chemotherapeutic resistance (Li et al., 2024). Consequently, Liang et al.'s findings showed that macrophages decrease the sensitivity of osteosarcoma cells to chemotherapy agents by secreting IL-1β during neoadjuvant chemotherapy, implying a potential decrease in chemotherapy efficacy in the osteosarcoma microenvironment (Liang et al., 2020). However, macrophages have been observed to exhibit anti-tumor functions under certain conditions. Gomez-Brouchet et al. indicated that elevated levels of CD163-positive macrophages in osteosarcoma are associated with improved prognosis, implying a positive influence on immune regulation in the TME (Gomez-Brouchet et al., 2017). Studies demonstrated that SKP2 deletion results in a proliferation of macrophages, correlating with a favorable prognosis in osteosarcoma patients, indicating a suppressive effect on tumor development and dissemination in the Rb1/p53-deficient mouse osteosarcoma model (Ferrena et al., 2023).

Withers et al. identified a correlation between enhanced CD204+ macrophage infiltration in canine osteosarcoma tissues and prolonged disease-free survival, underscoring macrophages’ potential advantageous impact in managing osteosarcoma progression (Withers et al., 2019). Within the osteosarcoma microenvironment, macrophages exhibit two polarization states: the pro-inflammatory M1 and the tumor-promoting M2. Richert et al. revealed the therapeutic potential of M1 macrophages in inhibiting tumor growth by promoting the conversion from M2 to M1 phenotype, offering insights into leveraging the tumor-suppressive capabilities of M1 macrophages (Richert et al., 2023). Pahl et al. observed that M1 macrophages, activated by LPS and IFN-γ, effectively suppress osteosarcoma cell proliferation, demonstrating an advantageous role in osteosarcoma immunotherapy (Pahl et al., 2014). Furthermore, Ji et al. confirmed that osteosarcoma tumor thrombi contain a substantial proportion of M1-like macrophages (TAM-M1), exhibiting an immunostimulatory state, positively influencing the activation of anti-tumor immune responses (Ji et al., 2023). Simultaneously, according to Gong et al., MPIRx nanoparticles prompt a specific mechanism causing macrophage polarization towards the M1 phenotype, thereby augmenting macrophage phagocytic function against osteosarcoma cells and contributing to an anti-tumor effect (Gong et al., 2023). Dumars et al. observed a substantial presence of M1 macrophages in non-metastatic osteosarcoma (OS Meta-), suggesting a potential role in impeding tumor metastasis (Dumars et al., 2016). Growing comprehension of TAMs underscores the potential for developing strategies that enhance immunotherapy effectiveness in osteosarcoma, focusing on the distinct polarization states of TAMs to advantageously impact tumor progression.

### 2.2 Myeloid-derived suppressor cell (MDSC)

In the TME, MDSCs are essential in suppressing the immune response. Predominantly, this suppression functions via interactions with T lymphocytes, resulting in the generation of reactive oxygen species (ROS) and the depletion of L-arginine. Such processes obstruct T cell proliferation and facilitate apoptosis, ultimately diminishing immune functionality (Joshi and Sharabi, 2022). Distinct MDSCs subtypes suppress T cell activity through diverse mechanisms: PMN-MDSCs primarily generate ROS via activation of STAT3 and upregulation of NADPH oxidase, while M-MDSCs predominantly produce nitric oxide (NO) by activating STAT1 and upregulating NO synthase, thus inhibiting T cell function. MDSCs attenuate acquired anti-tumor immunity alongside diminishing innate anti-tumor responses. In addition to T cells, MDSCs also suppress the functions of NK cells and DCs (Yang et al., 2020). Additionally, comprehensive research demonstrates that MDSCs instigate T cell inactivation through increased PD-L1 expression, interacting with PD-1 on T cells (Noman et al., 2014). CD155 expression on MDSCs enhances MDSCs-mediated T cell suppression, with significant reduction of MDSCs immunosuppressive activity *in vitro* using anti-TIGIT antibodies against TIGIT/CD155 pathway (Wu et al., 2019). These results collectively underscore that immune checkpoint molecules on MDSCs adversely regulate T cell function.

Specifically, Fan et al. demonstrated an innovative Metal-Organic Framework (MOF) nanosystem in treating osteosarcoma, offering concurrent chemo-immunotherapy and targeting MDSCs alongside modifying IDO activity, thereby substantially reducing tumor progression through a focus on the critical function of MDSCs (Fan et al., 2022). Additionally, the SDF-1/CXCR4 axis in the osteosarcoma microenvironment was found to contribute to MDSCs accumulation, diminishing the effectiveness of PD-1 therapy, and resulting in MDSCs infiltration that impeded the proliferation of cytotoxic T cells, demonstrating MDSCs’ adverse role in osteosarcoma (Jiang et al., 2019). Guan et al. identified a positive correlation between IL-18 and MDSCs accumulation in osteosarcoma, potentially facilitating MDSCs migration and tumor infiltration. Combining anti-IL-18 with anti-PD1 therapy effectively reduced levels of G-MDSC and M-MDSC, enhancing T cell infiltration and immune function, providing a new strategy for osteosarcoma immunotherapy (Guan et al., 2017). Shi et al. found a significant increase in MDSCs in the peripheral blood of osteosarcoma patients, with these cells promoting tumor immune evasion by inhibiting T cell activation and infiltration, thereby obstructing effective immune responses (Shi et al., 2019). Long et al. observed that MDSCs in the osteosarcoma TME significantly impede the function of GD2-CAR T cells, thus impairing the efficacy of these cells in osteosarcoma treatment and highlighting a negative immunoregulatory role of MDSCs in osteosarcoma (Long et al., 2016). Uehara et al. revealed that metformin significantly reduces abundance of MDSCs in osteosarcoma, favorably contributing to tumor growth control, highlighting the negative influence of MDSCs in osteosarcoma (Uehara et al., 2019). Post-neoadjuvant chemotherapy, Deng et al. noted a decrease in the quantity of HLA-DR-CD33^+^ MDSCs. Given their role in suppressing immune responses within the TME, this reduction might enhance the immune response against the tumor (Deng et al., 2020). Lastly, Kansara et al. showed that infiltrating myeloid cells in osteosarcoma influence IL23 through GRM4, contributing to tumor progression and indicative of a poor prognosis (Kansara et al., 2019).

### 2.3 T Cells

T lymphocytes, comprising the second most prevalent infiltrating cell type within the osteosarcoma TME, are functionally classified into cytotoxic T lymphocytes (CTLs), helper T lymphocytes (Th cells), and regulatory T cells (Tregs) (Itahashi et al., 2022). Overall, T lymphocytes exhibit a considerable heterogeneity and play a pivotal role in the immunotherapy of osteosarcoma. Notably, tumor-infiltrating lymphocytes (TILs) have been detected in 75% of osteosarcoma cases, with a higher incidence of 86% observed in metastatic osteosarcoma (Heymann et al., 2019).

Research has highlighted the essential role of CD8^+^ T cells and additional T cell subsets in osteosarcoma treatment. Investigations by Qi et al., through single-cell RNA sequencing, uncovered that low expression of GPR65 in osteosarcoma is prognostically unfavorable, and that its expression is positively connected with the enhanced activity and infiltration of immune cells, especially CD4^+^ and CD8^+^ T cells, that obstruct osteosarcoma progression (Qi et al., 2024). Subsequent investigations revealed that inhibition of c-Myc in osteosarcoma augmented the tumor immune microenvironment by elevating T cell chemokines and stimulating the CD40/CD40L pathway, facilitating the infiltration and activation of CTLs, highlighting the positive contribution of T cells in osteosarcoma treatment (Jiang et al., 2022). In addition, research conducted by Liu et al. elucidated modifications in the osteosarcoma immune microenvironment, specifically the perturbation of peripheral blood T lymphocyte subsets during osteosarcoma development, characterized by an increase in γδ T cells and Tregs, a decrease in CD8^+^ T cells, concomitant with peripheral immune suppression (Liu Q. et al., 2022). Ligon et al. underscored the distinctive response of T cells in the metastatic osteosarcoma TME, particularly at the tumor-lung interface, where T cells, resembling TILs, manifested significant immune suppression, with increased expression of immunosuppressive markers including PD-1 and LAG-3 (Ligon et al., 2021). Helm et al. observed a significant increase in CD8^+^ T cell infiltration in non-directly irradiated tumor areas following combined carbon ion radiotherapy and immune checkpoint inhibitor treatment, emphasizing the critical role of CD8^+^ T cells in limiting tumor growth and reducing lung metastases (Helm et al., 2021). Research by Yahiro et al. also indicated that activation of the TLR4 signaling pathway enhances the infiltration of CD8 positive cytotoxic lymphocytes in osteosarcoma lung metastases, effectively inhibiting osteosarcoma growth and metastasis, further confirming the importance of CD8^+^ cells in suppressing osteosarcoma progression (Yahiro et al., 2020). The infiltration of CD8 positive lymphocytes was significantly associated with improved survival in osteosarcoma patients treated with zoledronic acid, suggesting a positive immunoregulatory role of CD8 positive lymphocytes in inhibiting osteosarcoma growth and metastasis (Gomez-Brouchet et al., 2017). Research by Tang et al. revealed that upregulation of T-synthase in osteosarcoma enhances CD8^+^ T cell proliferation and function, thereby inhibiting tumor growth, while CD4^+^ T cells exert a suppressive effect on tumor cells through IFN-γ secretion (Tang et al., 2023). Moreover, the combined administration of IDO inhibitors and platinum(IV) prodrugs initiated the cGAS-STING pathway, enhancing CD8^+^ T cell function, which correlated with a more effective immune response and chemotherapy outcome, demonstrating potential in inhibiting tumor proliferation and dissemination (Xiang et al., 2023). Similarly, upregulation of miR-140 promoted increased infiltration of CD8^+^ T cells in the TME, directly related to the inhibition of osteosarcoma growth, suggesting a positive significance of increased CD8^+^ T cells in enhancing tumor immune response (Ji et al., 2018). However, the immunosuppressive environment introduces challenges to treatment. Research by Schell et al. in an SV40 large T antigen-induced osteosarcoma model found CTLs exhibited reduced responsiveness to particular epitopes, with a substantial reduction in specific CD8^+^ T cells, denoting tumor progression is associated with a decrease in immune cell efficacy, impacting tumor immune surveillance and response capabilities (Schell et al., 2000). Das et al. further emphasized the potential antitumor role of T cells in the osteosarcoma microenvironment, noting a positive correlation between T cell abundance and certain immune-related genes, such as PD-L1 and CD160 (Das et al., 2021). Additionally, Cillo et al. highlighted the suppressive effect of the TME on CD8^+^ T cell function, particularly through the expression of co-inhibitory receptors such as PD-1, revealing mechanisms of immune cell functional exhaustion in osteosarcoma recurrence (Cillo et al., 2022). Research by Liu et al. found that upregulation of microRNA-200a in osteosarcoma led to PD-L1-induced suppression of CD8^+^ T cell function, highlighting its potential as an immunotherapy target (Liu et al., 2020). Moreover, elevated levels of IL-35 in osteosarcoma patients inhibited the antitumor ability of CD8^+^ T cells, with Treg-secreted IL-35 related to the suppression of CD8^+^ T cell function (Liu MX. et al., 2019). Wang et al. discovered that Vγ9Vδ2 T cell activation enhanced the ability of CD8^+^ T cells to counter osteosarcoma, with CD8^+^ T cells managing osteosarcoma progression through the direct elimination of tumor cells (Wang S. et al., 2023). Wang et al. also observed that elevated PLOD2 expression in osteosarcoma was associated with enhanced CD8^+^ T cell infiltration, with such infiltration contributing positively to tumor suppression (Wang Z. et al., 2022). Concurrently, Cascio et al. noted that the expression levels of PD-L1 and HVEM in tumors were positively correlated with adjacent T cell infiltration, emphasizing the pivotal function of T cells in tumor immune surveillance (Cascio et al., 2021). Regardless of the challenges of immune escape and immunosuppression, emerging research has revealed strategies to tackle osteosarcoma by stimulating or enhancing T cell function. For instance, Shi et al. observed that the PI3Kδ/γ inhibitor SNA, by reducing MDSCs activity, improved the TME and augmented the effectiveness of anti-PD1 therapy, especially stressing the crucial role of CD8^+^ T cells’ activation in osteosarcoma (Shi et al., 2019). Furthermore, inhibition of the PD-1/PD-L1 pathway enabled CTLs to effectively attack osteosarcoma cells, contributing to tumor growth and metastasis suppression (Lussier et al., 2015). Similarly, the CD103+ cDC1s vaccine enhanced CD8^+^ T cell infiltration and activation in tumors, thereby inhibiting tumor growth and metastasis (Zhou et al., 2020b). Wang et al. demonstrated that in osteosarcoma treatment, sorafenib significantly inhibited the increase in PD-L1 expression induced by doxorubicin, thereby promoting the activity and proportion of CD8^+^ T cells in the TME, further confirming the significance of CD8^+^ T cells in resisting osteosarcoma (Wang et al., 2023b). Overall, studies consistently demonstrate the critical importance of T lymphocytes, particularly CD8^+^ T cells, in osteosarcoma treatment, highlighting their influence in impeding tumor development and metastatic development within a complex immunosuppressive environment.

### 2.4 Regulatory T cells (Tregs)

In osteosarcoma’s tumor immune microenvironment, Tregs have been identified as crucial, especially in mechanisms of immune escape. While contributing to immune tolerance, Tregs may be utilized by tumor cells, impeding effective anti-tumor responses and thus emphasizing Tregs’ significance in both tumor biology and immunotherapeutic research (Cheng D. et al., 2023). Elevated levels of microRNA-200a in osteosarcoma, correlating with an increased presence of Tregs (Foxp3+), imply Tregs actively contributing to immune evasion and tumor growth (Liu et al., 2020). Additionally, Li et al. revealed Tregs enhance the immunosuppressive microenvironment in osteosarcoma through Gal9 secretion and activation of the Tim3/Gal9 signaling pathway, thereby reducing the efficacy of Th1-type immune responses and enhancing immune escape mechanisms (Li et al., 2017). Addressing Tregs’ immunosuppressive influence in osteosarcoma, Kohyama et al. utilized an anti-IL-2 monoclonal antibody, S4B6, to deplete Tregs, eliciting an autologous immune response against osteosarcoma cells, significantly reducing both tumor size and lung metastases, underlining the importance of Tregs reduction in amplifying osteosarcoma’s immune response and its antitumor impact (Kohyama et al., 2012). Similarly, Kawano et al. utilized a dendritic cell and anti-GITR antibody combination to reduce Tregs levels, consequently enhancing the immune response to osteosarcoma (Kawano et al., 2015). Subsequent research has substantiated Tregs’ role in facilitating tumor cell immune evasion. Notably, Yoshida et al. reported that anti-PD-1 antibodies reduced Tregs infiltration in osteosarcoma, correlating with tumor growth delay and prolonged survival (Yoshida et al., 2020). Biller et al.'s research on canine osteosarcoma demonstrated an increase in Tregs and a concurrent reduction in the CD8^+^ T cells to Tregs proportion, correlating with diminished survival times, further emphasizing the importance of Tregs in facilitating tumor immune escape (Biller et al., 2010). Cheng et al. established that in osteosarcoma, Tregs drive tumor progression by activating pathways such as oxidative phosphorylation, angiogenesis, and mTORC1, in addition to their interactions in the TME (Cheng D. et al., 2023). Conclusively, Tregs play a pivotal role in facilitating immune evasion and tumor progression in osteosarcoma, establishing a basis for therapeutic strategies targeting Tregs and introducing novel approaches for osteosarcoma treatment.

### 2.5 Natural killer (NK) cells

NK cells, innate immune cells possessing cytokine production and cytotoxic capabilities, have been identified within the TME of osteosarcoma alongside their role in cytotoxicity and supporting T-cell activation. Specifically, NK cell-mediated IFN-γ release plays a crucial role in the activation of CD4^+^ T cells and is vital for the proliferation of CD8^+^ T-cell precursors (Myers and Miller, 2021). Preclinical investigations have shown the capability of NK cells to efficiently eliminate osteosarcoma cells (Razmara et al., 2021).

Notably, the expression of USP6 in Ewing’s sarcoma has been shown to facilitate NK cell invasion and activation, thereby enhancing their tumor cell-killing potential and underscoring the positive impact of NK cell infiltration in impeding tumor growth (Jain et al., 2023). Furthermore, USP6 also stimulates the secretion of immune-related chemokines, augmenting the infiltration and activation of NK cells and additional immunocytes, significantly impeding Ewing’s sarcoma progression (Henrich et al., 2021). Regarding the treatment of osteosarcoma, NK cells have emerged as crucial in controlling tumor progression and metastasis through their ability to recognize and eliminate cancer cells, affirming their infiltration and activation as beneficial in osteosarcoma mitigation (Kisseberth and Lee, 2021). The administration of Valproic Acid (VPA) has been demonstrated to elevate MICA/B expression on osteosarcoma cell surfaces, thus strengthening the NK cells’ cytotoxic activity and further emphasizing their critical role in osteosarcoma cell eradication (Yamanegi et al., 2010). NKT cells, resembling NK cells, significantly contribute to osteosarcoma inhibition. Kansara et al. highlighted the restraining effect of NKT cell infiltration on osteosarcoma development, induced by an immune response during cell senescence regulated by the RB1 gene (Kansara et al., 2013). iNKT cells, recognizing CD1d molecules on tumor cell surfaces, trigger cytotoxic responses that eliminate tumor cells and concurrently enhance the effectiveness of chemotherapeutic agents (Fallarini et al., 2012). Further investigations have revealed that the activation of IL-15 significantly augments the NK cell-mediated eradication of chemotherapy-resistant osteosarcoma cells, an effect dependent on the DNAM-1 and NKG2D pathways, indicating the specificity of NK cells in recognizing and eliminating osteosarcoma cells (Buddingh et al., 2011). Similarly, blocking FSTL1 has been found to strengthen the immune response of NK cells, effectively suppressing osteosarcoma cells and highlighting the advantageous impact of NK cell activation in tumor therapy (Ogiwara et al., 2022). In summary, NK cells play a critical role in the immunosurveillance and suppression within the osteosarcoma TME. Elevating the activation and function of such cells could significantly improve therapeutic efficacy against osteosarcoma, providing pivotal insights for advancing cancer therapy approaches.

### 2.6 Dendritic cells (DCs)

DCs are essential in the immune response, recognizing and presenting tumor antigens to helper and cytotoxic T cells and transitioning from immature to mature states, a key process for T cell activation; however, with osteosarcoma progression, tumor cells develop DC resistance, leading to diminished DC activation and consequent immune escape (Le et al., 2021). Specifically, CD103+ cDC1s have been identified as playing a crucial role in T cell activation and enhancing anti-tumor immune responses, particularly when combined with ICIs, exhibiting notable effectiveness against osteosarcoma (Zhou et al., 2020b). Additionally, tumor lysate-pulsed DC vaccines effectively activate the immune system, inhibiting osteosarcoma growth (Kawano et al., 2015), while DCs pulsed with EWS/FLI-l peptide epitopes exhibit significant capabilities in tackling Ewing’s sarcoma (Peng et al., 2014). Radiofrequency ablation (RFA) combined with OK-432 injection significantly increases intratumoral amounts of DCs, affirming the critical role of DCs in inducing distal immune effects and anti-tumor immune responses (Iwai et al., 2020). Moreover, the combined application of oncolytic adenovirus XVir-N-31, driven by YB-1, with CDK4/6 inhibitors, is observed to facilitate the maturation of monocyte-derived DCs, thus increasing their capacity in activating tumor antigen-specific T cells (Schober et al., 2023). However, as osteosarcoma advances, osteosarcoma cells develop resistance mutations against DCs and macrophages, resulting in decreased stimulation of DC activation and ultimately immune escape (Le et al., 2021). Research by Gao et al. revealed that in osteosarcoma patients, DC function is negatively regulated by miR-133a, an increase in which impairs DC maturation and activation, thereby promoting immune escape and tumor growth (Gao et al., 2016). In the immune microenvironment of osteosarcoma, regulatory DCs recruit Tregs, forming an immunosuppressive microenvironment that further facilitates tumor cells to evade immunosurveillance, assisting in tumor proliferation and dissemination (Liu et al., 2023). In summary, DCs exhibit a multifaceted yet crucial role in the immune response to osteosarcoma, capable of counteracting the tumor through immune activation but also potentially leading to immune escape due to resistance mechanisms in the TME. These findings provide critical evidence for potential therapeutic strategies targeting DCs, underscoring the significance of considering the modulation of DC functions in tumor therapy.

**FIGURE 1:**
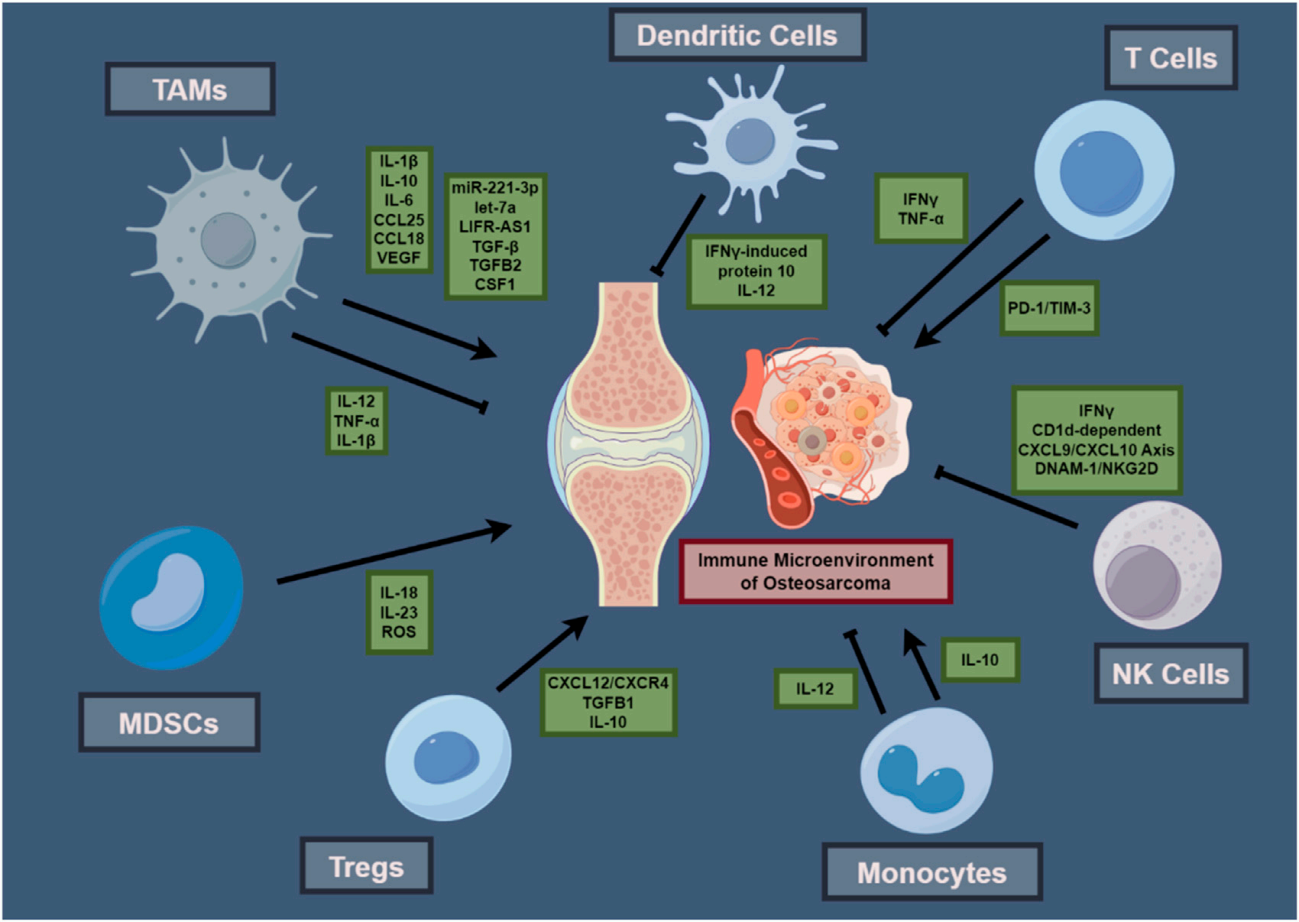
Interactions within the Osteosarcoma Tumor Immune Microenvironment.This schematic illustrates the interplay among immune cells and their cytokines within the osteosarcoma microenvironment, featuring TAMs, DCs, T cells, MDSCs, Tregs, monocytes, and NK cells, with their cytokines—ILs and TNFs—central to their function in the tumor’s immunology. Arrows suggest interactive influences and potential immunotherapeutic targets, summarizing principal components that contribute to osteosarcoma’s immunological functioning and treatment response.

In conclusion, the immune microenvironment within osteosarcoma presents a complex landscape characterized by an intricate network of cell signaling mechanisms, regulatory pathways, and cellular interactions. The central functions of DCs, in conjunction with T cells, macrophages, MDSCs, Tregs, and NK cells, converge to influence tumor progression, immune surveillance, and the efficacy of therapeutic interventions. The interplay between immunosuppressive activity and antitumor immunity is determined by cellular dynamics, affecting the tumor’s ability for immune evasion and the effectiveness of immunotherapeutic interventions. The intricate interactions and interactive contributions of immune cells within the osteosarcoma TME, which underscore the investigated mechanisms, are concisely depicted, presenting a diagrammatic elucidation that enhances comprehension of such intricacies (Figure 1). To further illustrate the multifaceted roles and therapeutic targets of these immune cells, Table 1 presents a comprehensive overview of immune cell attributes in the osteosarcoma tumor microenvironment, detailing their diverse functions and roles as therapeutic targets, thereby enriching the understanding of tumor development and immune regulation.

**TABLE 1:** Characteristics of the osteosarcoma tumor immune microenvironment.

Immune cell type	Therapeutic target/Role	Impact on tumorigenesis and immune regulation	References
CD163(+) M2-Type TAMs	Amplification of T cell-specific immunosuppression	Facilitate osteosarcoma progression by inhibiting T cell activity, with depletion improving T cell response	Han et al. (2016)
CD11b+ Myeloid Cells (MDSCs and TAMs)	Metformin reprograms immune cell metabolism, bolstering anti-tumor activity	Metformin reduces MDSCs and shifts TAMs from an M2-like immunosuppressive to an M1-like pro-inflammatory phenotype, thereby inhibiting osteosarcoma growth	Uehara et al. (2019)
M2-Type TAMs	ZIM3 upregulation in osteosarcoma activates CCL25, driving M2 migration	M2 TAMs’ recruitment to lung metastases potentiates osteosarcoma metastatic aggression	Li et al. (2023)
TAMs	MerTK-mediated efferocytosis in macrophages facilitates M2 polarization and PD-L1 expression	M2 polarization and PD-L1 expression in macrophages foster immune tolerance, promoting osteosarcoma advancement	Lin et al. (2022)
M2 Macrophages	Induced by osteosarcoma-derived exosomes via Tim-3 signaling	M2 macrophage polarization contributes to the metastatic spread of osteosarcoma by promoting tumor cell migration and EMT	Cheng et al. (2021)
FABP4+ Macrophages	FABP4+ Macrophages’ presence in metastatic lesions underscores their function in osteosarcoma metastasis	FABP4+ Macrophages’ proinflammatory state at metastatic sites enhances osteosarcoma’s metastatic capacity	Zhou et al. (2020a)
CD163-Positive TAMs, CD8-Positive Cytotoxic Lymphocytes	CD163-Positive TAMs as biomarkers for survival prognosis; CD8-Positive Cells as indicators for response to zoledronate treatment	High levels of CD163-Positive TAMs correlate with better survival outcomes; presence of CD8-Positive Cells associated with improved survival in zoledronate-treated patients	Gomez-Brouchet et al. (2017)
MDSCs	Targeting CXCR4 to inhibit MDSCs’ immunosuppressive function and enhance anti-PD-1 therapy	MDSCs mediated resistance to anti-PD-1 therapy in osteosarcoma via CXCR4/SDF-1 pathway and AKT activation	Jiang et al. (2019)
GRM4-Expressing Myeloid Cells	GRM4 in myeloid cells is pivotal for IL23 expression, contributing to osteosarcoma’s malignancy	Myeloid cells via GRM4-dependent IL23 regulation promote osteosarcoma growth, with IL23 blockade or GRM4 agonism reducing tumor development	Kansara et al. (2019)
CTLs	Targeting MYC with JQ-1 to Foster CTL Recruitment and Enhance Antigen-Presenting Cell-T Cell Crosstalk via CD40/CD40L	Enhanced CTL presence and activity within the tumor microenvironment, leading to reduced tumor burden and improved survival in osteosarcoma	Jiang et al. (2022)
CTLs	LPS-induced TLR4 activation promotes CD8^+^ T cell infiltration and function in osteosarcoma lung metastases	Enhanced CD8^+^ CTL activity through TLR4 leads to reduced tumor volume and improved survival in osteosarcoma models	Yahiro et al. (2020)
Human Vγ9Vδ2 T cells	Vγ9Vδ2 T cells act as antigen-presenting cells (APCs), effectively priming CD8^+^ T cells for antitumor responses	Through the HSP90-MyD88-JNK signaling pathway, Vγ9Vδ2 T cells enhance antigen presentation, leading to potent CD8^+^ T cell-mediated tumor suppression	Wang et al. (2023a)
CD103+ conventional dendritic cells (cDC1s)	In vitro-generated CD103+cDC1s act as potent antigen-presenting cells, exhibiting superior antigen cross-presentation and T cell priming capabilities	CD103+cDC1 vaccination curtails tumor growth by stimulating T cell activity, with improved results when used alongside CTLA-4 inhibitors	Zhou et al. (2020b)
Tregs	Tregs enhance osteosarcoma immunosuppression through CXCL signaling, affecting CXCR4 and TGFB1	Tregs impact osteosarcoma through oxidative phosphorylation, angiogenesis, and mTORC1 signaling, with genes CD320 and MAF critical in these pathways and as prognostic indicators	Cheng et al. (2023a)
Tim3+ T cells and monocytes; Gal9+ Tregs	Interaction between Tim3 and Gal9 dampens T cell activity and shifts monocyte cytokine secretion, suppressing Th1 responses	Interactions between Tim3+ T cells and Gal9-expressing cells lead to a dampened Th1 response, contributing to the immunosuppressive tumor microenvironment in osteosarcoma	Li et al. (2017)
NK cells	USP6 in Ewing sarcoma stimulates NK cell cytotoxicity	USP6 not only facilitates NK cell-mediated tumor suppression at the primary site but also stimulates a broad NK cell response, curbing the growth of metastatic tumors	Jain et al. (2023)
Dendritic cells and T cells	The combined treatment with XVir-N-31 and CDK4/6 inhibitors enhances the maturation of dendritic cells and activates tumor-specific T cell responses	Enhanced immune cell activity against the tumor results in reduced tumor growth and increased survival rates	Schober et al. (2023)

## 3 Osteosarcoma treatment

### 3.1 Targeted therapies

Regarding osteosarcoma treatment, current advancements have highlighted the capacity of varied targeted treatment strategies to counteract this malignancy effectively. Liao et al.’s investigation into sulfatinib, a novel tyrosine kinase inhibitor targeting FGFR1, CSF1R, and VEGFR1-3, revealed its efficacy in curtailing osteosarcoma cell proliferation and invasion. The inhibitory effects are attributed to a dual-action mechanism that encompasses direct tumor cell suppression and modulation of the TME. The study delineates the capacity of sulfatinib to diminish M2 macrophage polarization and reduce populations of M2-TAMs, Tregs, and MDSCs, concurrently amplifying cytotoxic T-cell presence, hence attenuating the immunosuppressive context of the TME (Liao et al., 2023). Subsequent research by Proença et al. indicated that cyanidin suppresses osteosarcoma cells by escalating Bax expression and caspase-3 activity, triggering the intrinsic apoptotic pathway, and concurrently decreasing the secretion of inflammatory cytokines like IL-6, IL-1β, and IL-12p70, illustrating its therapeutic potential in osteosarcoma (Proenca et al., 2023). Wang et al. synthesized a nanomedicine that targets osteosarcoma-specific molecular markers precisely, utilizing the self-assembly of ROS and esterase-activated linker-connected cabazitaxel (diCTX) with diDHA, which induces significant cytotoxicity in the TME upon activation (Wang et al., 2023c). Similarly, Dieudonné et al. enhanced the sensitivity to the chemotherapeutic drug doxorubicin by targeting the Wnt/TCF signaling pathway, emphasizing the importance of molecular targeted therapy in osteosarcoma treatment (Dieudonne et al., 2012). Research by Martins-Neves et al. demonstrated that IWR-1, by inhibiting the Wnt/β-catenin signaling pathway, diminishes the self-renewal capability of osteosarcoma cancer stem cells, consequently reducing tumor growth and chemoresistance, highlighting the potential of targeting the Wnt/β-catenin pathway in osteosarcoma treatment strategies (Martins-Neves et al., 2018). Zheng et al. identified that soy isoflavones suppress the AKT/mTOR pathway, initiating mitochondrial autophagy, which in turn impedes osteosarcoma progression at a molecular level (Zheng Z. et al., 2024). Yu et al.’s approach in treating pulmonary metastatic osteosarcoma involves engineered hepatic genetic circuits for efficient VEGFR2 siRNA delivery, directly targeting tumor cells, diminishing adverse drug reactions, and potentially improving immune cell functionality (Yu et al., 2023). Furthermore, research by Cheng et al. revealed that Psoralidin inhibits osteosarcoma growth and metastasis by targeting the FAK and PI3K/Akt signaling pathways and downregulating ITGB1 expression (Cheng S. et al., 2023). Regarding molecular mechanisms, Wu et al. indicated that FGD1 promotes tumor progression by inhibiting PTEN activity, with overexpression associated with poor prognosis in osteosarcoma patients, activating the PI3K/AKT signaling pathway, and affecting tumor cell behavior, suggesting FGD1 as a potential target for improving osteosarcoma treatment outcomes (Wu et al., 2020). Higuchi et al. demonstrated that the combination of pioglitazone and cisplatin is an effective strategy for molecular targeted therapy in osteosarcoma, significantly inhibiting tumor growth by targeting PPARγ (Higuchi et al., 2020). Clinical trials have also shown promising results. The study by Kopp et al. conducted a clinical trial of glembatumumab vedotin (GV) in patients with recurrent osteosarcoma, revealing that GV exhibits antitumor activity in the treatment of recurrent osteosarcoma (Kopp et al., 2019). Research by Gaspar et al. similarly indicated that the combined use of lenvatinib, etoposide, and ifosfamide demonstrated promising antitumor activity in patients with recurrent or refractory osteosarcoma, warranting further exploration in randomized phase II studies (Gaspar et al., 2021). The application of targeted therapies in osteosarcoma treatment, by inhibiting tumor growth, modulating the microenvironment, and enhancing drug response, introduces novel directions for treatment strategies and contributes to a more thorough perception of therapeutic mechanisms.

### 3.2 Immunotherapy for osteosarcoma

#### 3.2.1 Immune checkpoint inhibitors

For osteosarcoma treatment, ICIs are considered promising due to the significantly higher quantity of TILs in osteosarcoma compared to other sarcomas (Wedekind et al., 2018). The increased TILs in osteosarcoma suggest potential for more effective utilization of ICIs (van Erp et al., 2017). Principal inhibitors include PD-1, PD-L1, and CTLA4, each applicable in osteosarcoma treatment (Yu et al., 2022). Liu et al. demonstrated that activation of the STAT3 pathway by Thrombospondin-1 (TSP1) results in the upregulation of PD-L1, subsequently inhibiting the immune response in osteosarcoma. Subsequent analyses showed that neutralizing antibodies against PD-L1 can mitigate TSP1’s suppressive impact on CD8^+^ T cells (Liu Z. et al., 2022). The findings of this study offer a foundation for comprehending TSP1’s contribution to immune suppression in osteosarcomas and developing PD-L1 centric therapeutic strategies. Additionally, Sorafenib was observed to inhibit the Doxorubicin-induced upregulation of PD-L1, thereby ameliorating the immunosuppressive microenvironment in osteosarcoma. The synergistic application of Sorafenib and Doxorubicin significantly suppressed tumor growth and increased the proportion of CD8^+^ CTLs secreting IFN-γ in the tumor tissue (Wang et al., 2023b). Such modulation of the immune microenvironment by these combined pharmaceuticals has been shown to augment the treatment efficacy for osteosarcoma. Furthermore, Sundara et al. established that in metastatic osteosarcoma lesions, there is an elevation in PD-L1 expression alongside augmented T cell infiltration, indicating that T cell-based immunotherapy might be beneficial for patients with metastatic osteosarcoma (Sundara et al., 2017). Subsequent investigations have further highlighted the role of ICIs in osteosarcoma therapy. Xiang et al. demonstrated that combining IDO inhibitors with a platinum (IV) prodrug activates the cGAS-STING pathway, which results in enhanced CD8^+^ T cell activity and an improved tumor immune microenvironment (Xiang et al., 2023). Correspondingly, Ji et al. conducted research on the function of miR-140 within the osteosarcoma immune microenvironment, revealing that its reduction of PD-L1 not only impedes tumor proliferation but also intensifies CD8^+^ T cell infiltration, thereby ameliorating the immune microenvironment (Ji et al., 2018). Research by Mochizuki et al. indicated that OBP-502, a telomerase-specific oncolytic virus, not only improves the efficacy of anti-PD-1 antibodies in osteosarcoma treatment but also induces immunogenic cell death in tumor cells through integrin binding, leading to an increase in calreticulin, ATP, and HMGB1, and enhancing CD8^+^ T cell infiltration in the tumor (Mochizuki et al., 2021). Similarly, the investigation conducted by Sung et al. revealed that ICSBP enhances PD-L1 expression in osteosarcoma cells, leading to increased cell proliferation, and showed that decreasing PD-L1 not only inhibits tumor cell expansion but also strengthens the impact of chemotherapeutic drugs, proposing new treatment modalities for osteosarcoma (Sung et al., 2022). Ahangar et al. further noted that silencing the immune checkpoint HHLA2 enhances the sensitivity of osteosarcoma cells to Paclitaxel, increasing its cytotoxic effects (Ahangar et al., 2023). Additionally, research has indicated that elevated PD-L1 mRNA expression in osteosarcoma tissues, along with inhibiting the PD-1/PD-L1 axis, improves Cisplatin’s therapeutic efficacy, and the concurrent administration of anti-PD-1 antibodies with Cisplatin markedly diminishes tumor cell growth and enhances apoptosis (Liu X. et al., 2019). However, in the treatment of osteosarcoma, overcoming immune evasion, particularly the overexpression of PD-L1 and diminished T cell function, remains a significant challenge. Research by Liu et al. indicated that microRNA-200a enhances PD-L1 expression through the upregulation of PTEN, subsequently suppressing the immune response and reducing the efficacy of CD8^+^ T cells, thereby promoting tumor growth and impacting the effectiveness of PD-L1 targeted immunotherapy (Liu et al., 2020). Studies suggest that specific inhibitors or combination therapies can effectively address these challenges. For instance, the specific inhibition of PI3Kδ/γ by SNA inhibitors was found to improve the TME and, in conjunction with anti-PD1 therapy, decelerates tumor progression while prolonging survival time (Shi et al., 2019). Additionally, Ge et al. discovered that CBZP nanocarriers, activating ICD and inhibiting PD-1/PD-L1 interactions, significantly enhanced the efficacy of PD-1/PD-L1 immune checkpoint blockade therapy. This nanotherapeutic strategy, incorporating CUR and BMS1166, modulates autophagy and improves the TME, increasing CD8^+^ T cell infiltration in osteosarcoma, thereby achieving notable anti-tumor effects (Ge et al., 2022). Subsequent studies have revealed that a combination approach using different ICIs, such as inhibitors targeting CTLA-4, PD-L1, and TIM3, is more efficacious in reducing osteosarcoma cell viability and inducing apoptosis relative to individual blockades (Sorkhabi et al., 2022). Moreover, the integration of high-energy carbon ion radiotherapy with ICIs (anti-PD-1 and anti-CTLA-4) has been shown to exert substantial therapeutic effects on osteosarcoma lung metastases, reducing the metastases while concurrently augmenting CD8^+^ immune cell infiltration in tumors not directly exposed to radiation (Helm et al., 2021). In osteosarcoma immunotherapy research, various strategies have been proposed to enhance treatment effects and overcome immune evasion mechanisms. Duan et al. revealed that sunitinib, by targeting STAT3 to suppress PD-L1 expression, remodels the immune landscape and impedes tumor cell migration and invasion. When utilized alongside ICIs, sunitinib substantially reduces lung metastasis and tumor proliferation, leading to improved survival rates (Duan et al., 2020). Furthermore, Kawano et al. proposed using anti-CTLA-4 antibodies and cryo-treated tumor lysate-loaded DCs in a mouse osteosarcoma model to enhance the anti-tumor immune response. This combination therapy effectively suppresses osteosarcoma lung metastasis and improves survival rates through the reduction of Tregs and an increase in CD8^+^ T cells (Kawano et al., 2013). Research by He et al. further emphasized the importance of combination therapy, finding that the combined treatment of L-arginine with α-PD-L1 antibodies enhances the immune system’s response to osteosarcoma. L-arginine promotes the proliferation, differentiation, and survival of CD8^+^ T cells, elevating serum levels of IFN-γ and CD8^+^ T cell infiltration, while α-PD-L1 antibodies protect these enhanced CD8^+^ T cells from exhaustion (He et al., 2017). Simultaneously, Yu et al. revealed that the combination of the autophagy inhibitor 3-MA with photodynamic therapy (PDT) exhibits potential, primarily through the suppression of PD-L1 protein expression, which in turn activates an immune response that is effective in suppressing the proliferation and dissemination of osteosarcoma cells (Yu et al., 2019). Zheng et al. examined the PD-1 pathway in musculoskeletal tumors, finding that while PD-L1 expression negatively correlated with osteosarcoma patient survival, PD-L2 and PD-1 expressions demonstrated positive and negative associations with survival, respectively; moreover, in humanized mouse models, nivolumab’s inhibition of osteosarcoma metastasis highlighted the PD-1 pathway as a promising target for immunotherapeutic intervention in metastatic osteosarcoma (Zheng B. et al., 2018). Further, Lussier et al. found that PD-L1 expression on metastatic osteosarcoma cells, through its interaction with PD-1 on CTLs, impaired CTL function; conversely, disruption of the PD-1/PD-L1 interaction substantially improved CTL functionality, reduced tumor burden, prolonged patient survival (Lussier et al., 2015). Additionally, Ratti et al. discovered that Trabectedin inhibits osteosarcoma through the promotion of osteosarcoma cell differentiation and an increase in tumor-infiltrating T cells. Owing to the elevated PD-1 expression on intratumoral CD8 T cells, the integration of PD-1 inhibitors with standard therapy significantly enhances therapeutic effectiveness (Ratti et al., 2017). The study by Gao et al. revealed that the PD-1/PD-L1 axis inhibits Tfh cell function and reduces IL-21 secretion in osteosarcoma patients, thus obstructing potent anti-tumor responses in osteosarcoma patients; conversely, the application of ICIs assists in restoring Tfh cell functionality, thereby enhancing CD8^+^ T cell efficacy (Gao et al., 2017). The application of ICIs in osteosarcoma treatment, particularly targeting key checkpoints such as PD-1, PD-L1, and CTLA-4, has been proven to effectively modulate the TME and stimulate the activity of TILs, thus eliciting a potent anti-tumor immune response, offering a novel perspective in the treatment of osteosarcoma.

#### 3.2.2 Tumor microenvironment modulators

Tumor microenvironment modulators in osteosarcoma immunotherapy have shown immense potential. Jiang et al. conducted a comprehensive analysis of single-cell samples from osteosarcoma, revealing the role of genes associated with aggregated autophagy, suggesting that interventions targeting aggregated autophagy may improve survival rates and quality of life for patients with osteosarcoma (Jiang et al., 2023). Huang et al. identified the significant immunomodulatory effects of Methionine Enkephalin (MENK) in osteosarcoma treatment. It was shown that MENK enhances the activity of M1 macrophages and reduces the proportion of M2 macrophages, thereby exerting an inhibitory effect on osteosarcoma. Furthermore, MENK also influences tumor-associated immune cells, such as the distribution of MDSCs, providing new research directions and potential therapeutic strategies for osteosarcoma immunotherapy (Huang et al., 2023). In the studies by Zhang et al., let-7a was confirmed to have a tumor-suppressive role in Ewing’s sarcoma, particularly through negatively regulating macrophage infiltration. By affecting the interactions between STAT3 and NF-κB, let-7a inhibits the recruitment of macrophages, thus reducing the malignancy of Ewing’s sarcoma (Zhang et al., 2016). Further research by Zhang et al. showed that EPA reduces DDP-induced high expression of PD-L1 and decreases immune evasion in osteosarcoma, a process that helps enhance the sensitivity of osteosarcoma to chemotherapy and may improve treatment outcomes (Zhang et al., 2023). Herrador-Cañete et al. demonstrated significant therapeutic effects in a pediatric osteosarcoma model by employing SFV-mediated Gal3 inhibitors, which not only weakened the binding of Gal3 to activated T cells but also inhibited tumor growth and pulmonary metastasis, thereby accentuating the significance of diminishing the tumor’s immunosuppressive environment by augmenting lymphocyte infiltration (Herrador-Canete et al., 2022). The study conducted by Denton et al. on Ewing’s sarcoma treatment highlighted the pivotal role of M2 macrophages in the TME in influencing tumor growth and response to therapy. Administering liposomal clodronate (Clodrosome) alongside trabectedin was proven to effectively diminishes M2 macrophage infiltration, thus amplifying the effect of HSV oncolytic virus therapy in Ewing’s sarcoma and emphasizing the critical necessity to regulate macrophage polarization states within the TME for improved therapeutic outcomes (Denton et al., 2018). Similarly, Wang et al. demonstrated the efficacy of combining curcumin with cisplatin in inhibiting tumor-associated macrophage polarization towards M2, enhancing cisplatin-induced apoptosis, effectively inhibiting osteosarcoma cell proliferation and migration (Wang J. et al., 2022). Zhou et al. revealed the potential of all-trans retinoic acid (ATRA) in reducing osteosarcoma metastasis by inhibiting M2 polarization of TAMs, further emphasizing the importance of regulating TAM polarization in osteosarcoma treatment (All-Trans Retinoic Acid Prevents, 2020). Belisario et al. elucidated the importance of ABCA1 and ABCB1 expression ratios in determining osteosarcoma cell sensitivity to chemotherapy and immunotherapy. Enhanced ABCB1 expression reduces intracellular doxorubicin accumulation, while increased ABCA1 expression bolsters Vγ9Vδ2 T cell activity. These findings imply that osteosarcoma cells with high ABCB1 and low ABCA1 expression are more resistant to chemotherapy and immunotherapy, with bisphosphonates potentially acting as sensitizing agents (Belisario et al., 2020). The research of Miallot et al. suggested that the vitamin B5 precursor pantothenamide enhances anti-tumor immunity in sarcomas, including promoting dendritic and myeloid cells toward an IFNγ-driven antigen presentation pathway, and fostering a high metabolic state in CD8^+^ T cells (Miallot et al., 2023). Wang et al. revealed that the combined application of valproic acid and zoledronic acid enhances γδ T cell-mediated cytotoxicity against osteosarcoma cells. The mechanism primarily involves the accumulation of methylhydroxybutyrate pathway intermediates and relies on T cell receptor-mediated granule exocytosis pathways (Wang S. et al., 2018). Gong et al. noted that the MPIRx nanomedicine, incorporating the sonosensitizer IR780 and CD47 inhibitor RRx-001 into PEG-PCL nanomicelles, effectively promotes macrophage polarization toward the M1 phenotype and enhances phagocytic activity against tumor cells, significantly inhibiting osteosarcoma and its pulmonary metastasis (Gong et al., 2023). Makielski et al. found that the oncolytic virus VSV-IFNβ-NIS is safe and effective in canine osteosarcoma, where VSV-treated tumors exhibited stronger inflammatory responses and increased expression of T cell immune gene clusters, enhancing anti-tumor immune responses and improving long-term survival rates; this study further emphasizes the potential of oncolytic viruses in activating immune responses within the TME (Makielski et al., 2023). Park et al.'s research on improving the TME demonstrated that VEGF inhibitors, such as bevacizumab and DC101, optimized tumor vasculature, enhancing EATs’ infiltration and cytotoxic impact in solid tumors. The study showed an increase in high endothelial venules and a significant promotion of T cell, especially CD8(+) TILs, infiltration into tumors, achieving notable anti-tumor effects in diverse xenograft tumor models without an increase in toxicity (Park et al., 2023). Wu et al. discovered that hydroxyapatite nanoparticles (HANPs) with varying aspect ratios enhanced immunogenic cell death in tumor cells, activated macrophages, and promoted the maturation of CD8^+^ T cells and DCs, with particles of higher aspect ratios exhibiting stronger anti-tumor activity (Wu et al., 2023). In a mouse model of Rb1/p53-deficient osteosarcoma, Ferrena et al. observed that SKP2 gene knockout resulted in increased immune cell infiltration and transcriptional changes associated with favorable prognosis. A notable increase in the expression of macrophage-specific genes was detected, positively influencing overall survival rates in osteosarcoma patients (Ferrena et al., 2023). Moreover, advancements in non-invasive modalities and targeted therapy strategies are under thorough investigation. Hay et al. demonstrated that the application of ultrasound technology was efficient in ablating tumors and inducing immune responses in the TME, as indicated by the proliferation of macrophages and DCs, and upregulation of genes associated with immune activation (Hay et al., 2023). In an osteosarcoma mouse model, Yahiro et al. demonstrated that activation of TLR4 markedly increased CD8-positive cell infiltration in lung metastases and inhibited osteosarcoma progression. Activation of TLR4 by LPS enhanced accumulation of CD8-positive cells in tumors, and its inhibition negated LPS’s tumor-suppressive effects, underscoring the pivotal role of CD8-positive cells in anti-tumor responses (Yahiro et al., 2020). Regarding targeted signaling pathways, Fujiwara et al. ascertained that Pexidartinib (PLX3397), a CSF1/CSF1R signaling inhibitor, reduced CSF1 secretion in osteosarcoma cells, leading to diminished polarization and chemotaxis of M2-type macrophages. Concurrently, it facilitated the infiltration of CD8-positive T cells at both primary and metastatic osteosarcoma sites, contributing to the inhibition of tumor progression and enhancement of patient prognosis (Fujiwara et al., 2021). Similarly, Li et al. indicated that osteosarcomas decrease CXCL12 expression via DNA methyltransferase 1 (DNMT1), impacting tumor metastasis and T cell infiltration. A positive correlation was observed between CXCL12 levels, intratumoral lymphocyte presence, and survival rates in patients. Inhibiting DNMT1 in a mouse model resulted in heightened CXCL12 expression, enhancing immune responses and impeding tumor metastasis and growth (Li et al., 2018). Specific immunotherapeutic strategies, such as those demonstrated by Richert et al., have shown significant antitumor effects in the treatment of osteosarcoma with the use of liposomal-encapsulated chemically detoxified lipopolysaccharides (Lipo-MP-LPS) acting as TLR4 agonists. The mechanism involves the conversion of M2-type macrophages to M1-type, altering the immune microenvironment of osteosarcoma to a pro-inflammatory state and enhancing the recruitment of T cells and antitumor immune responses (Richert et al., 2023). Further investigations have revealed that particular therapeutic approaches, including radiofrequency ablation, nanomedicine delivery, and oncolytic virotherapy, can also enhance the activity of specific immune cells within the TME. Studies by Iwai et al., which combined radiofrequency ablation with OK-432 injections, induced systemic antitumor effects primarily characterized by increased numbers of specific immune cells, such as DCs and CD8^+^ T cells, and the promotion of pro-inflammatory cytokines (IFN-γ and TNF-α) within the tumor, revealing the potential of combined physical and chemical interventions in modulating the TME (Iwai et al., 2020). Additionally, Zhang et al. employed self-stabilizing hyaluronic acid nanoparticles for targeted delivery of anticancer drugs, such as docetaxel and cisplatin, along with the immunostimulatory agent resiquimod, promoting immunogenic cell death and immune activation within the TME, which significantly inhibited tumor growth in osteosarcoma mouse models (Zhang Y. et al., 2021). Research by Li et al. found that osteosarcoma cell-secreted LGALS3BP binds to LGALS3 on the surface of M1-type macrophages, inducing secretion of HSPA1L through the Akt phosphorylation pathway, promoting the transformation of M1-type macrophages to an antitumor phenotype, and inducing tumor cell apoptosis through the IRAK1 and IRAK4 pathways (Li et al., 2022). Moreover, studies by Jiang et al. have shown that high expression of c-Myc in osteosarcoma is inversely correlated with T cell infiltration rates. The c-Myc inhibitor JQ-1 significantly reduced tumor burden and increased survival rates in mouse models of osteosarcoma by promoting T cell migration to the tumor and activating specific CTLs through the CD40/CD40L costimulatory pathway, thereby improving the tumor immune microenvironment (Jiang et al., 2022). Research by Uehara et al. revealed that metformin could inhibit osteosarcoma growth by affecting the metabolic and functional characteristics of CD11b+ cells, particularly by reducing the number of MDSCs and altering the phenotype of TAMs, suggesting that modulating the metabolic state of specific immune cells in the TME can achieve tumor growth inhibition (Uehara et al., 2019). Furthermore, studies by Jain et al. observed that USP6 inhibits tumor growth in Ewing’s sarcoma by activating NK cells and inducing the expression of chemokines. USP6 increases the surface levels of NK cell activation ligands, enhancing the interaction between NK cells and Ewing’s sarcoma cells, thus augmenting the tumor-killing capability of NK cells and highlighting the significance of USP6 in regulating the TME (Jain et al., 2023). Research by Pahl et al. emphasized that LPS and IFN-γ-activated M1-type macrophages could inhibit osteosarcoma growth, with a similar effect observed when M1-type macrophages were activated with L-MTP-PE and IFN-γ. Additionally, IL-10-induced polarized M2-type macrophages could inhibit osteosarcoma growth in the presence of anti-EGFR cetuximab through an antibody-dependent mechanism, demonstrating the potential of macrophages in osteosarcoma treatment (Pahl et al., 2014). Interventions targeting the microenvironment of osteosarcoma have demonstrated potential for therapy, exerting direct impacts on neoplastic cells and bolstering immune responses, thereby providing possibilities for improved treatment outcome.

#### 3.2.3 Cytokine therapy

In the exploration of new strategies for osteosarcoma treatment, cytokine therapy has exhibited substantial potential. Specifically, IL-2 cytokine therapy in canine osteosarcoma is crucial for the immune response, as it stimulates T-cell activation and proliferation, thus substantially improving tumor cell identification and elimination (Flesner et al., 2020). The results of this study are in concordance with those presented by Hennessy et al., who demonstrated that BEMPEG (NKTR-214), as an IL-2 pathway agonist, delayed tumor progression and enhanced survival rates in murine models of osteosarcoma (Hennessy et al., 2021). Furthermore, Nastasi et al. established that the inhibition of IL-10 through combined administration of anti-IL-10 antibodies and Mifamurtide substantially increased tumor cell mortality and decreased metastasis in osteosarcoma treatment, emphasizing the significance of IL-10 regulation in immune cells and its potential to augment the effectiveness of Mifamurtide therapy (Nastasi et al., 2023). Concurrently, Rebhun et al. investigated the application of inhaled recombinant human interleukin-15 (rhIL-15) in the treatment of lung metastases in canine osteosarcoma and melanoma. Their findings suggested that diminished lymphocyte levels before treatment and heightened peak cytotoxicity were significantly associated with therapeutic effectiveness, demonstrating rhIL-15’s considerable clinical potential in inhibiting tumor metastasis (Rebhun et al., 2022). Morales-Molina et al. utilized a combined immunotherapeutic approach involving mesenchymal stem cells (MSCs) bearing tumor-specific oncolytic adenoviruses (OAds) and granulocyte colony-stimulating factor (G-CSF) in osteosarcoma models, substantially reducing tumor growth; it was observed that G-CSF improved infiltration and activity of CD8^+^ T cells and DCs in the tumor immune microenvironment, effectively enhancing tumor immunosurveillance and elimination while diminishing T cell exhaustion (Morales-Molina et al., 2021). In summary, cytokine therapy in osteosarcoma treatment has shown potential by enhancing immune cell function and reducing immune escape, offering new perspectives for therapy.

#### 3.2.4 Tumor antigen-specific therapies

The specificity of therapies targeting tumor antigens has proven valuable in cancer treatment, particularly in osteosarcoma research, demonstrating the potential of enhancing treatment efficacy through targeting tumor antigens. Park et al. demonstrated that the use of dual-specificity antibodies targeting GD2 and HER2 (T-BsAb) significantly enhanced osteosarcoma treatment outcomes, with these antibodies substantially inhibiting tumor growth in both *in vitro* and *in vivo* models; the addition of anti-PD-L1 treatment further augmented their antitumor activity (Park and Cheung, 2020). Moreover, Wiebel et al. revealed an association between cell density and GD2 expression in osteosarcoma cells, finding that increased cell density enhanced the reactivity to GD2-specific CAR T cells, providing a basis for optimizing CAR T therapy (Wiebel et al., 2021). In the search for innovative immunological targets, Li et al. discerned two novel CTL epitopes derived from the osteosarcoma antigen papillomavirus binding factor (PBF) with immunostimulatory potential in the HLA-A11 context (Li et al., 2019). Watanabe et al. developed tumor-associated antigen-specific T cell receptor (TCR) multimers, particularly PBF TCR multimers, demonstrating high efficiency in recognizing naturally presented peptides and effectively identifying tumor-associated antigens on osteosarcoma cells, thus introducing novel strategies for immunotherapy (Watanabe et al., 2019). Research conducted by Tsukahara et al. further confirmed PBF as a significant tumor-associated antigen in osteosarcoma, providing novel peptide-based immunotherapeutic targets, with its extensive presence in osteosarcoma cells and tissues highlighting its potential for immunotherapeutic applications. Cytotoxic T lymphocyte clones’ recognition of specific PBF peptides emphasized their importance in therapy (Tsukahara et al., 2004). Additionally, the identification of novel epitopes has propelled advancements in oncological therapies. Osei-Hwedieh et al. demonstrated that T cell recognition of these epitopes is imperative for tumor immunosurveillance, with sarcomas, particularly osteosarcomas, exhibiting a range of antigenic profiles conducive to T cell-mediated immunotherapy enhancement (Osei-Hwedieh et al., 2023). Tsuda et al. identified the presence of SART3 antigen in osteosarcoma and showed that peptides derived from SART3 could activate specific cytotoxic T cells to target HLA-A24 positive osteosarcoma cells, presenting novel molecular targets for immunotherapy against osteosarcoma (Tsuda et al., 2001). In developing therapies targeting specific markers, Englisch et al. discovered that VEGFR2 is prevalently expressed in tumor-associated vascular endothelial cells of Ewing’s sarcoma and engineered CARs targeting VEGFR2 with variable hinge lengths, which, when expressed in human T cells, specifically lysed VEGFR2-expressing target cells through mechanisms including enhanced antigen-specific degranulation, effective tumor spheroid lysis, TNF-α secretion, sustained killing, and cellular proliferation, indicating VEGFR2 as a potent target for CAR T cell therapy in Ewing’s sarcoma (Englisch et al., 2020). Specificity in tumor antigen-targeted therapies has made significant strides in osteosarcoma research, effectively targeting tumor antigens with specific antibodies, CAR T cells, and T cell receptor multimers, revealing new immunological targets and therapeutic strategies, providing novel insights for treatment.

#### 3.2.5 Tumor vaccines

Significant progress has been made in the field of osteosarcoma treatment, particularly in vaccine development. Domingo-Musibay et al.'s findings indicated engineered measles virus (MV) as an effective agent against osteosarcoma cells, demonstrating substantial oncolytic impacts, including tumor growth reduction and increased survival durations in osteosarcoma xenograft mouse models (Domingo-Musibay et al., 2014). Additionally, Mason et al. investigated the recombinant *Listeria* ADXS31-164, expressing HER2/neu fusion protein, and their results validated its effectiveness in activating tumor-specific immune responses in canine osteosarcoma models, notably enhancing IFNγ responses, effectively reducing metastasis rates, and improving survival outcomes (Mason et al., 2016). Advancements in autologous cancer cell vaccine research were notable, as illustrated by Flesner et al., who employed these vaccines effectively in treating canine osteosarcoma, demonstrating their significant therapeutic impact by activating the canine immune system against the tumor, thereby underscoring the importance of personalized treatment and the potential of utilizing the autologous immune system in tumor management (Flesner et al., 2020). Furthermore, Peng et al. demonstrated that DCs, when pulsed with EWS/FLI-l peptides, effectively induced immune responses in Ewing’s sarcoma mouse models, not only triggering IFN-γ secretion from effector cells but also specifically targeting and eradicating specific tumor cells (Peng et al., 2014). Adenovirus and DNA vaccines have also shown therapeutic potential. Schober et al. revealed that the integration of YB-1 driven adenovirus XVir-N-31 with CDK4/6 inhibitors elevated the immunogenicity of Ewing’s sarcoma, enhancing both viral replication and oncolytic capacity, and, through the activation of HLA-I and IFNγ-inducible protein 10, it enhanced DCs maturation and specific T cell activation against tumor antigens (Schober et al., 2023). Tarone et al. demonstrated that the HuDo-CSPG4 DNA vaccine’s role in activating CD8^+^ T cells and anti-CSPG4 immune responses, leading to the induction of CD8^+^ T cells and serum that hinder osteosarcoma growth and metastasis, concurrently confirming its safety and survival-prolonging effects in canine osteosarcoma models (Tarone et al., 2023). Regarding dendritic cell vaccines, Zhou et al. demonstrated that CD103+ cDC1s vaccines, particularly when augmented with anti-CTLA-4 antibodies, significantly enhanced control over melanoma and osteosarcoma, completely eradicated osteosarcoma tumors, and effectively prevented lung metastasis (Zhou et al., 2020b). Miwa et al. established the safety of DC vaccine therapy in eliciting immune responses in patients with refractory sarcomas, acknowledging that, although clinical responses varied, DC vaccines demonstrated potential in enhancing anti-tumor immune responses (Miwa et al., 2017). Immunomodulators have shown promise in the treatment of osteosarcoma. Research by Cascini et al. revealed that local injections of TLR9 agonists in osteosarcoma mouse models significantly inhibited tumor growth and elicited notable effects in untreated contralateral tumors. These applications led to substantial changes in the immune microenvironment, including a reduction in M2-type macrophages, increased infiltration of DCs, and the activation of CD8 T cells, suggesting the potential of TLR9 agonists as *in situ* anti-tumor vaccines (Cascini et al., 2023). Wang et al. developed calcium phosphate nanoparticles for *in situ* vaccination against osteosarcoma, engineered to control the release of chemotherapeutic agents and immunomodulators, thus activating DCs and CD8^+^ T cells, and effectively inhibiting tumor progression and metastasis (Wang Y. et al., 2023). Additionally, Pritchard-Jones et al. discovered that the 105AD7 vaccine effectively elicited T cell responses and specific IFN-γ secretion in osteosarcoma patients post-intensive chemotherapy, underscoring its potential in immunoregulatory therapy (Pritchard-Jones et al., 2005). In osteosarcoma treatment, Chauvin et al. developed a novel vaccine based on rat-derived killer dendritic cells (KDCs), which, by activating KDCs to target and eliminate tumor cells and effectively present tumor antigens *in vivo*, induced a strong CD8 T cell-mediated immune response (Chauvin et al., 2008). Moreover, Jin et al. demonstrated that capsaicin induced calreticulin (CRT) exposure on human osteosarcoma cell surfaces, initiating immunogenic cell death (ICD) and augmenting the phagocytic capabilities of antigen-presenting cells along with lymphocyte IFN-γ secretion, contributing to the experimental foundation for tumor vaccine strategies in osteosarcoma (Jin et al., 2016). Cumulatively, these studies illustrate the potency of vaccines in precisely targeting and eliminating osteosarcoma through the stimulation of the immune system, advancing understanding of the tumor’s immune interactions and suggesting innovative directions for forthcoming treatments.

#### 3.2.6 Cell-based therapies

Cell-based therapies represent a significant advancement in osteosarcoma treatment, particularly in the domain of chimeric antigen receptor T-cell (CAR-T cell) therapy. Charan et al. revealed that combining HGF receptor-neutralizing antibodies (AMG102) with GD2-targeted CAR-T cell therapy inhibited tumor growth and metastasis in Ewing’s sarcoma, offering an effective treatment option for tumors with hyperactive HGF/c-MET pathways (Charan et al., 2020). Furthermore, Evans et al.’s exploration of EWS-FLI-1 in Ewing’s sarcoma family of tumors (ESFT) revealed that modified EWS-FLI-1 peptides notably activated CTLs against Ewing’s sarcoma, demonstrating significant cytotoxicity *in vitro* against ESFT and effectively suppressing tumor growth in mouse models (Evans et al., 2012). Another study, conducted by Hidalgo et al., presented evidence that FITC-labeled anti-B7-H3 antibodies used for controlled activation of CAR T cells targeting cancer cells enhanced specificity of the therapy, decreased side effects on normal tissues, and effectively treated osteosarcoma in animal models (Hidalgo et al., 2023). Research by Fallarini et al. found that invariant natural killer T cells (iNKT cells) in osteosarcoma therapy exerted cytotoxicity on osteosarcoma cells via a CD1d-dependent mechanism and augmented cell death caused by cisplatin, doxorubicin, and methotrexate (Fallarini et al., 2012). Additionally, according to Buddingh et al., IL-15 activated NK cells present a novel therapeutic approach against high-grade osteosarcoma, including cells resistant to chemotherapy (Buddingh et al., 2011). Kailayangiri et al. confirmed that Ewing’s sarcoma cells uniformly express G(D2) antigens, offering a theoretical rationale for applying CAR T cell immunotherapy, with GD2-targeted CAR T cells exhibiting high specificity and cytotoxicity against tumor cells, thus suggesting a novel approach for Ewing’s sarcoma treatment (Kailayangiri et al., 2012). The investigation by Mensali et al. revealed that ALPL-1 targeted CAR-T cells had significant impact in osteosarcoma treatment, selectively recognizing and destroying ALPL-positive osteosarcoma cells and exhibiting notable anti-tumor activity in in situ models, all while maintaining normal tissue safety (Mensali et al., 2023). Long et al., in their exploration of GD2-CAR T cells for osteosarcoma, revealed significant cytotoxic potential of these genetically engineered T cells against GD2ww-positive tumor cells, with the study highlighting the TME, notably the presence of MDSCs, as a potential barrier to efficacy, and recommending all-trans retinoic acid to reduce MDSCs and improve therapeutic outcomes (Long et al., 2016). In addition, a study highlighted the efficacy of CAR T cell therapy against CD166 in osteosarcoma, with CD166.BBζ CAR-T cells demonstrating significant cytotoxicity *in vitro* against osteosarcoma cells, and mouse models further confirming their effectiveness in tumor growth inhibition with minimal toxicity (Wang et al., 2019). Treatments based on γδ T cells and NK cells also showed therapeutic potential. Wang et al.’s findings indicated that human Vγ9Vδ2 T cells, as antigen-presenting cells, hold potential in osteosarcoma treatment; their activation increased CCL5 secretion through the upregulation of HSP90 production, MyD88, and JNK activation, effectively enhancing CD8^+^ T cell responses against osteosarcoma cells (Wang S. et al., 2023). A further study indicated that decitabine, a demethylating agent, amplified the expression of NKG2D ligands including MICB and ULBP1 on osteosarcoma cell surfaces, thus rendering them more susceptible to γδ T cell attack. Blocking the NKG2D receptor partly reversed the γδ T cell-induced cytotoxicity, suggesting a significant reliance on the NKG2D-NKG2DL axis, and highlighted the possibility of enhanced efficacy with combined decitabine and γδ T cell therapy (Wang Z. et al., 2018). Kisseberth et al. conducted research using autologous NK cell transplantation aimed at overcoming cancer-induced reductions in NK cell quantity and functionality, offering innovative treatment options for canine osteosarcoma (Kisseberth and Lee, 2021). Significant attention has been focused on the utilization of iPSC-derived CTLs and memory T cells in cell therapy applications. Ishii et al. demonstrated that iPSC-derived rejTs, focusing on neoantigens from the EWS/FLI1 fusion gene, induced notable immune effects against Ewing’s sarcoma. In co-culture with Ewing’s sarcoma cell lines, rejTs consistently exhibited suppressive effects on tumor cells, and the anti-tumor capacity of EWS/FLI1-rejT, along with enhanced survival rates, was further verified in mouse xenograft models of Ewing’s sarcoma (Ishii et al., 2021). Fernández et al. indicated that CD45RA-memory T cells, expressing the NKG2D-4-1BB-CD3z CAR, targeted osteosarcoma cells expressing NKG2D ligands effectively, with these cells showing greater cytotoxicity against osteosarcoma cells, which points to the essential role of immune cells, notably memory T cells, in focused osteosarcoma therapy (Fernandez et al., 2017). Additionally, some studies have focused on enhancing therapeutic efficacy by modifying PBMCs and incorporating immunomodulators. Yang et al. introduced a membrane-anchored and tumor-targeted IL12-PBMC therapy with profound effects on osteosarcoma. This strategy, through PBMC modification, eliminated the traditional CAR T therapy’s expansion phase and enhanced tumor-specific cytokine release, leading to effective inhibition of tumor growth (Yang et al., 2022). Hu et al. demonstrated that attIL12-T cells, equipped with membrane-anchored tumor-targeted IL-12, specifically engaged osteosarcoma cells through interaction with carapatin (CSV) on the tumor surface. This interaction led to increased IFNγ production, reduced TGF-β activity, apoptosis induction in CAFs, and disruption of the ECM, thereby facilitating T cell infiltration, enhancing immune cell function, and inhibiting tumor growth (Hu et al., 2024). Cell therapy, especially CAR-T technology, has introduced innovative approaches in osteosarcoma treatment, while advancements in iNKT, NK cells, and iPSC-derived cells have extended treatment methodologies, enhancing both specificity and effectiveness.

#### 3.2.7 Antibodies

In osteosarcoma treatment research, the application of antibodies has shown significant potential for immunomodulation. Initially, Kohyama et al. revealed that employing the monoclonal antibody S4B6, which targets IL-2, resulted in significant inhibition of tumor growth in osteosarcoma models in mice. S4B6 operates by depleting Tregs and activating autoimmune responses, with its administration at multiple time points around LM8 cell transplantation consistently diminishing tumor size and lung metastases (Kohyama et al., 2012). Subsequently, Kawano et al. established that the combined deployment of agonistic anti-GITR antibodies with DCs enhanced immune responsiveness in osteosarcoma therapy, reducing the presence of Tregs and effectively suppressing tumor proliferation. This dual therapy approach not only increased CD8^+^ T cell levels and IFN-γ but also reduced immunosuppressive factors within the tumor, introducing an innovative method for osteosarcoma immunotherapy (Kawano et al., 2015). Additionally, the combined administration of anti-TGF-β antibodies and DCs in a mouse model for osteosarcoma significantly enhanced systemic immune responses, thereby restricting metastatic tumor growth by boosting CD8^+^ T cell levels and reducing the prevalence of Tregs (Kawano et al., 2012). The combined therapy of CD47 mAb and doxorubicin, as shown by Mohanty et al., effectively increased macrophage and other immune cell infiltration in osteosarcoma, resulting in substantial tumor growth suppression (Mohanty et al., 2019). These research findings demonstrate that antibody application in osteosarcoma treatment not only directly suppresses tumor growth but also adjusts crucial cell types and immune responses, offering innovative strategies and approaches for osteosarcoma therapy.

Investigating immunotherapeutic strategies in osteosarcoma reveals crucial interventions modifying tumor-immune interactions. Table 2 presents targeted immune cells, corresponding treatments, their mechanisms, and immune consequences, emphasizing the principal aspects of therapy and their impacts on immune regulation.

**TABLE 2:** Overview of immunotherapeutic strategies in osteosarcoma.

Treatment category	Immune cell type	Therapeutic agent	Therapeutic mechanism and immune regulation effects	References
Immune Checkpoint Inhibitors	CD8^+^ T cells	Anti-PD-L1 antibodies	TSP1-mediated PD-L1 expression suppresses CD8^+^ T cell activity in osteosarcoma, reversed by PD-L1 antagonists	Liu et al. (2022b)
Immune Checkpoint Inhibitors	T cells	Anti-PD-L1/HLA class I agents	Increased T cells in metastases with HLA class I and PD-L1 suggest immunotherapy potential	Sundara et al. (2017)
Oncolytic & Immune Checkpoint Therapy	CD8^+^ T Cells	Telomerase-specific oncolytic virus (OBP-502) and PD-1 inhibitors	Oncolytic virus OBP-502 increases PD-L1 and immunogenic signals, bolstering CD8^+^ T cell efficacy and PD-1 inhibitor response in osteosarcoma	Mochizuki et al. (2021)
Immune Checkpoint Inhibitors & Autophagy Inhibition	CTLs	ZnPc/BSA (PDT) and 3-MA (Autophagy Inhibitor)	Combining ZnPc/BSA-PDT with 3-MA downregulates PD-L1, boosting CTLs’ anti-tumor activity in osteosarcoma	Yu et al. (2019)
Immune Checkpoint Therapy	CD4^+^ and CD8^+^ T Cells	Nivolumab (a PD-1 blocking antibody)	Nivolumab boosts CD8^+^ T cell efficacy against osteosarcoma metastasis	Zheng et al. (2018b)
Combined Cellular and Antibody-Based Therapy	BsAb-Armed CD8^+^ TILs	VEGF Inhibitors (Bevacizumab, DC101) and BsAbs	Improving CD8^+^ TILs’ tumor infiltration and action by normalizing tumor vasculature, amplifying BsAb-mediated T cell antitumor responses	Park et al. (2023)
Immunomodulation via Nanotechnology	CD8^+^ T Cells, Macrophages	Hydroxyapatite Nanoparticles (HANPs)	HANPs enhance CD8^+^ T cell and macrophage-mediated tumor immunity, especially effective with higher aspect ratio nanoparticles	Wu et al. (2023)
Epigenetic Immunomodulation	CD8^+^ T Cells	DNMT1 epigenetic inhibitors for CXCL12 upregulation	Inhibiting DNMT1 elevates CXCL12, bolstering CD8^+^ T-cell engagement and anti-tumor efficacy in osteosarcoma	Li et al. (2018)
Cytokine Therapy	T Cells	Autologous Vaccine, T-Cell Transfer, IL-2	Vaccine and IL-2-boosted T cells effectively combat osteosarcoma, extending survival by amplifying immune attack on cancer cells	Flesner et al. (2020)
Cytokine Therapy	Lymphocytes	Inhaled rhIL-15	rhIL-15 enhances lymphocyte-mediated attack on osteosarcoma metastases	Rebhun et al. (2022)
Tumor Antigen-Specific Therapies	T cells	Bispecific antibodies for GD2/HER2	Targeted antibodies against GD2/HER2 augment T cell accuracy and the elimination of osteosarcoma	Park and Cheung (2020)
Tumor Antigen-Specific Therapies	CTLs	Papillomavirus Binding Factor (PBF) derived peptides	CTL-mediated recognition of PBF peptides enhances targeting and destruction of osteosarcoma cells	Tsukahara et al. (2004)
Tumor Vaccines	T cells	HER2/neu-targeting *Listeria* (ADXS31-164)	T cell activation by the HER2/neu-targeting ADXS31-164 vaccine led to reduced metastasis and increased survival in canine osteosarcoma	Mason et al. (2016)
Tumor Vaccines	CD8^+^ T cells	CSPG4-specific chimeric DNA vaccine	Activation of CD8^+^ T cells by the CSPG4 vaccine resulted in decreased osteosarcoma growth and enhanced survival	Tarone et al. (2023)
Cell-based Therapies	CAR-T cells	GD2-directed CAR-T cells and HGF-targeted neutralizing antibody (AMG102)	CAR-T cells, aided by HGF antibody AMG102, targeted and reduced Ewing sarcoma growth and spread	Charan et al. (2020)
Cell-based Therapies	CAR-T cells	ALPL-1-targeted CAR-T	Targeting ALPL-1, CAR-T cells showed effective anti-osteosarcoma activity in preclinical tests	Mensali et al. (2023)
Cell-based Therapies	CAR-T cells	Anti-CD166/4-1BB CAR-T cells	CD166-directed CAR-T cells effectively targeted osteosarcoma, bolstered by 4-1BB’s role in T cell improvement	Wang et al. (2019)
Cell-based Therapies	iPSC-derived rejuvenated CTLs (rejTs)	EWS/FLI1-specific rejTs	EWS/FLI1-specific rejTs from iPSCs showed potent anti-Ewing sarcoma activity	Ishii et al. (2021)
Antibodies	Dendritic cells, CD8^+^ T cells	Anti-GITR antibody, dendritic cells	Anti-GITR with dendritic cells suppressed osteosarcoma, lowered regulatory T cells, and increased CD8^+^ T cells, improving immune response against the tumor	Kawano et al. (2015)
Antibodies	Dendritic cells, CD8(+) T cells	Dendritic cells with anti-TGF-β	Combining dendritic cells with anti-TGF-β increased CD8(+) T cells, decreased regulatory T cells, and hindered osteosarcoma metastasis by improving immune response	Kawano et al. (2012)

## 4 Conclusion and perspectives

This review comprehensively outlines significant advancements in the field of osteosarcoma treatment, with particular emphasis on the potential of novel therapies such as ICIs, tumor microenvironment modulators, cytokine therapy, and targeted treatments against specific antigens. Developments in CAR-T cell therapy alongside the employment of iPSC-derived CTLs and memory T cells represent a paradigm shift in cancer treatment strategies towards personalized and targeted approaches. The integration of antibodies further enhances these therapeutic strategies, improving specificity and effectiveness.

Despite these advancements, several limitations remain. A primary impediment is the intricacy of the TME and its role in immune evasion. The heterogeneity of osteosarcoma cells and disparate treatment outcomes among distinct patient groups also pose substantial hurdles. Moreover, numerous novel therapies are still in developmental or clinical trial phases, with their long-term efficacy and safety yet to be fully established. More rigorous clinical trials are required to validate the effectiveness of these therapies across diverse patient demographics.

In the future, sustained investigation is imperative to surmount these obstacles and to enrich comprehension of the complex interplays between osteosarcoma cells and the immune mechanism. Subsequent studies should focus on developing more effective combination therapies that can synergistically enhance treatment outcomes. Personalized medicine approaches, utilizing genomic and proteomic profiling, could offer more tailored and effective treatment strategies. The exploration of novel immunotherapeutic targets and the optimization of current treatment modalities are crucial for improving patient outcomes. Finally, fostering multidisciplinary collaborations will be essential in accelerating the translation of these scientific insights into clinical practice, providing new hope for patients with osteosarcoma.

## Abbreviations

TME, Tumor Microenvironment; TAMs, Tumor-Associated Macrophages; MDSCs, Myeloid-Derived Suppressor Cells; NK cells, Natural Killer Cells; DCs, Dendritic Cells; IDO, Indoleamine 2,3-Dioxygenase; PD-1, Programmed Cell Death Protein 1; CTLs, Cytotoxic T Lymphocytes; Th cells, Helper T Lymphocytes; Tregs, Regulatory T Cells; TILs, Tumor-Infiltrating Lymphocytes; ICIs, Immune Checkpoint Inhibitors; CAR-T, Chimeric Antigen Receptor T-Cell; PBMCs, Peripheral Blood Mononuclear Cells; G-CSF, Granulocyte Colony-Stimulating Factor; TCR, T Cell Receptor; iNKT cells, Invariant Natural Killer T Cells.
